# SARS-CoV-2 pneumonia succesfully treated with cpap and cycles of tripod position: a case report

**DOI:** 10.1186/s12871-020-01221-5

**Published:** 2021-01-08

**Authors:** Michela Rauseo, Lucia Mirabella, Rosa Roberta Caporusso, Leonarda Pia Cantatore, Marco Paolo Perrini, Paolo Vetuschi, Daniela La Bella, Livio Tullo, Gilda Cinnella

**Affiliations:** Department of Anesthesia and Intensive Care Unit, Università degli Studi di Foggia, Azienda Ospedaliero Universitaria “Ospedali Riuniti di Foggia”, Viale Pinto, 1, 71122 Foggia, Italy

**Keywords:** COVID-19, SARS-CoV-2 pneumonia, Helmet CPAP, Tripod position

## Abstract

**Background:**

Pneumonia induced by 2019 Coronavirus (COVID-19) is characterized by hypoxemic respiratory failure that may present with a broad spectrum of clinical phenotypes. At the beginning, patients may have normal lung compliance and be responsive to noninvasive ventilatory support, such as CPAP. However, the transition to more severe respiratory failure - Severe Acute Respiratory Syndrome (SARS-CoV-2), necessitating invasive ventilation is often abrupt and characterized by a severe V/Q mismatch that require cycles of prone positioning. The aim of this case is to report the effect on gas exchange, respiratory mechanics and hemodynamics of tripod (or orthopneic sitting position) used as an alternative to prone position in a patient with mild SARS-CoV-2 pneumonia ventilated with helmet CPAP.

**Case presentation:**

A 77-year-old awake and collaborating male patient with mild SARS-CoV-2 pneumonia and ventilated with Helmet CPAP, showed sudden worsening of gas exchange without dyspnea. After an unsuccessful attempt of prone positioning, we alternated three-hours cycles of semi-recumbent and tripod position, still keeping him in CPAP. Arterial blood gases (PaO2/FiO2, PaO2, SaO2, PaCO2 and A/a gradient), respiratory (VE, VT, RR) and hemodynamic parameters (HR, MAP) were collected in the supine and tripod position. Cycles of tripod position were continued for 3 days. The patient had a clinically important improvement in arterial blood gases and respiratory parameters, with stable hemodynamic and was successfully weaned and discharged to ward 10 days after pneumonia onset.

**Conclusions:**

Tripod position during Helmet CPAP can be applied safely in patients with mild SARS-CoV-2 pneumonia, with improvement of oxygenation and V/Q matching, thus reducing the need for intubation.

## Background

Hypoxemic respiratory failure is the characteristic aspect for presentation of pneumonia induced by 2019 Coronavirus (COVID-19). SARS-CoV-2 patients present with a wide spectrum of clinical severity, ranging from asymptomatic to pneumonia to ARDS-like phenotypes [1, 2]. In the making of more robust evidence, widely shared hypothesis suggests that together with viral load and patient’s physiological reserve, the activation’s amplitude of two biological cascades, the interleukin 6 (IL-6) cytokine storm [3] and the disseminated intravascular cascade (DIC) [4], are responsible for the inflammatory host response in the lungs.

At the beginning, patients may have normal lung compliance and be responsive to noninvasive ventilatory support, such as CPAP. However, the transition to more severe respiratory failure - Severe Acute Respiratory Syndrome (SARS-CoV-2), necessitating invasive ventilation is often abrupt and characterized by a severe V/Q mismatch that require cycles of prone positioning [2, 5].

**Fig. 1:**
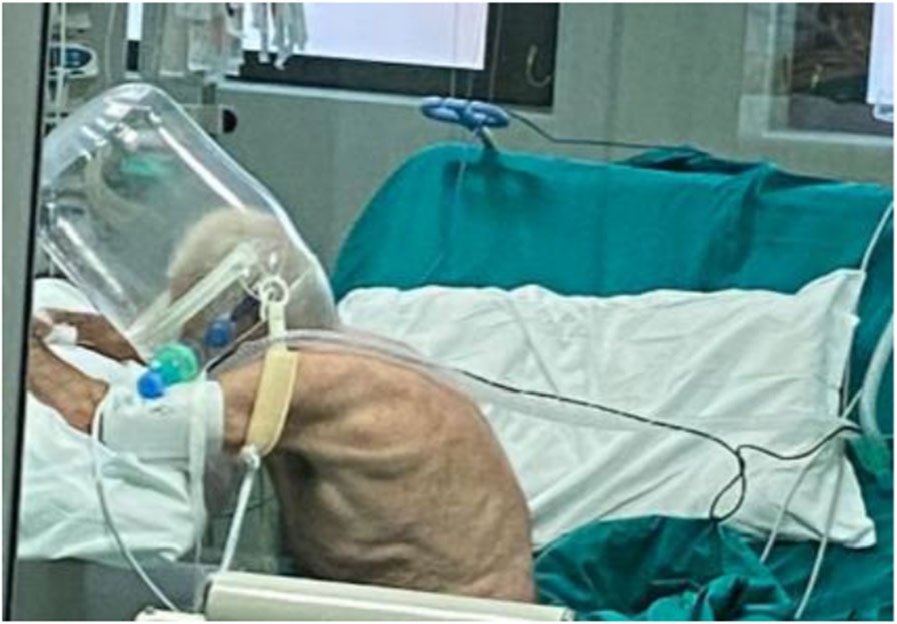
Patient in tripod position during Helmet CPAP

We report on the clinical course of a patient suffering from refractory hypoxemia due to COVID-19 pneumonia treated with CPAP and helmet interface in the out of bed tripod position (Fig. 1). We choose this solution, since the patient was well adapted to CPAP, but did not tolerate other facial interfaces nor prone position. Repeated shifting from supine to tripod determined a stable improvement of ventilation to perfusion (V/Q) matching and PaO2/FiO2 ratio and prevented intubation and invasive mechanical ventilation.

## Case presentation

A 77-year-old man was admitted to the emergency department (ER), on March 6th. His symptoms commenced on the previous day, while at home, with sore throat, dry cough, mild dyspnea and subjective fevers. He had been in contact in earlier days with an individual positive for COVID-19 disease, and was otherwise healthy. Physical examination revealed a temperature of 37 °C, pulse rate of 80 beat/min, mean blood pressure (mBP) 80 mmHg, respiratory rate (RR) 18 breath/min and oxygen saturation 87% in room air. SARS-CoV-2 was detected in a nasopharyngeal swab specimen by real-time reverse-transcriptase PCR. After having unsuccessfully been treated for 24 h with Venturi Mask Oxygen in the Infectious Diseases department, the patient was referred to our ICU and CPAP with helmet immediately started.

At ICU admission he had no fever, WBC 4.35*10^3^ mcL and CRP of 120 mg/L. Chest X ray showed interstitial lung infiltrates, no sign of fatigue nor tachypnea.

**Fig. 2:**
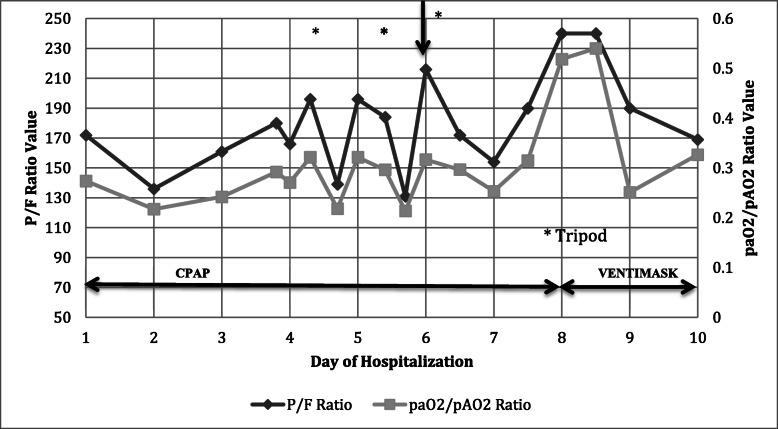
Time course of recorded P/F ratio and paO2/pAO2 ratio during CPAP with alternating cycles of semirecumbent and tripod position. The worst data recorded daily are reported

On admission CPAP was set at 10 cmH_2_O with a FiO_2_ of 50%, in order to reach a VT of 6–7 ml/Kg PBW. Under such setting, the highest PaO2/FiO2 was 172, PaCO2 34 mmHg, pH 7.34, RR 20. On march 8th, PaO2/FiO2 decreased to 136 and CPAP was increased to 12 cmH2O, with FiO2 55%. PaO2/FiO2 further decreased and helmet was then temporarily substituted with a full face mask in order to position him prone. However, both face mask and prone position were not tolerated. The patient was awake and responsive, and in a last attempt to avoid intubation he was asked to assume an orthopneic (or tripod) position (Fig. 1). Rapidly, after the assumption of tripod position SaO2 improved and patient was kept in the same position with CPAP 10 cmH2O and FiO2 55%. The PaO2/FiO2 ratio increased from 136 to 196 (*p* < .05) after 3 h and PaO2/PAO2 went from 0.22 to 0.34, showing an actual recovery in terms of O2 effective delivery (Fig. 2). The respiratory rate decreased from 20 to 17 (Table 1) and we were able to lower CPAP to 8 cmH2O and FiO2 to 40%, without changes in SaO2 or hemodynamics. The patient felt more comfortable and able to take some rest in that position compared to the supine position. We continued alternating cycles of tripod and semirecumbent positions for the following 4 days (Fig. 2). Eight days after ICU admission, he was weaned successfully to a conventional O2 Ventimask. SARS-CoV-2 was detected in nasopharyngeal swab specimen until March 28th. On day 10, with a resolution of the pneumonia, he was sent back to a post-COVID ward isolation and then discharged home.

**Table 1:** Respiratory mechanics and hemodynamics in both positions

	Supine	Tripod	***p*** value
PEEP (cmH2O)	10	10	=1
SaO2 (%)	93	99	*< 0.05*
VT (ml)	370	420	*< 0.05*
VE (L/min)	5.6	7.2	=0.2
RR (bpm)	20	17	=0.14
PaO2/FiO2	136	196	*< 0.05*
FiO2 (%)	50	50	=1
A-a(O2) (mmHg)	244	232	*< 0.05*
PaO2/PAO2	0.22	0.34	=0.89
mBP (mmHg)	90	80	=0.4
HR (bpm)	60	60	=1

## Discussion and conclusions

To our knowledge this is the first report of refractory hypoxemia successfully treated with CPAP with helmet and cycles of tripod position. Moreover, this case novelty in our opinion lies also on the peculiar pathology treated, i.e. SARS-CoV-2 pneumonia.

Patients requiring hospitalization usually have hypoxemia due to increased lung permeability, loss of perfusion’s regulation with hyper-perfusion of inflamed lung areas and significant ventilation to perfusion (V/Q) mismatch due to shunt effect, more than to atelectasis [2, 5].

At the beginning, hypoxemia may be compensated by an increase in minute ventilation without marked dyspnea. To some extent in such cases the inspiratory effort is not disproportionate and work of breathing (WOB) tolerable. At this stage, patients are responsive to an increase in FiO_2_ and to non-invasive ventilation approaches, such as Continuous Positive Airways Pressure (CPAP), aimed at maintaining alveolar patency and improving V/Q matching. However, the transition to more severe clinical patterns of acute respiratory failure is often very rapid. When hypoxemia persist or worsen, the inspiratory load may become excessive, generating too negative swings of intrathoracic pressure and further lung injury [6–8]. Under such conditions intubation should not be delayed. Clinicians have thus barely a very narrow windows to profit of before shifting to invasive mechanical ventilation when patients are unresponsive to non-invasive approaches.

Hence, changing patients’ position can allow for taking advantage of this “no man’s land” to obtain pulmonary blood flow redistribution, better V/Q matching and increased PaO2/FiO2 ratio.

Actually, body position is not a neutral aspect of patients’ care, as evidenced by an amount of physiological, experimental and clinical studies in different surgical and ICU settings [9, 10]. The modification in position can affect respiratory mechanics by changing resistance and/or compliance of the respiratory system and its lung and chest wall components, and by changing static lung volume and its regional distribution. Mechanical ventilation, either invasive or non-invasive, is commonly delivered in a semi-recumbent supine position, that offer the advantage of increased Functional Residual Capacity (FRC), reduced airways resistance and WOB and improved oxygenation versus the supine [11, 12]. Prone positioning (PP) classically is being used in severe respiratory failure to reopen collapsed lung areas, obtain a more even tidal volume distribution together with a redistribution of pulmonary blood flow [10–12], while in SARS-COV-2, PP is indicated mainly because of its effects on blood flow [2, 6–8].

Under such conditions, the orthopneic or tripod position is a middle way. This posture is often assumed instinctively by individuals experiencing shortness of breath and used in dyspneic patients that sit and lean forward with hands on their knees or on the side of the bed with an over bed table in front to lean on and several pillows on the table to rest on. Patients experience subjective relief of dyspnea, while diaphragmatic function and thoraco-abdominal movements are improved, thus allowing a better V/Q matching [9–14], as in the present case. Recently, Ding et al. [15] reported the first cases of mild ARDS under non-invasive ventilation successfully treated with PP, without need for intubation. We here suggest that in patients not tolerating to be prone, an alternative may be to keep them in tripod.

In conclusion, to our knowledge, this is the first case that report successful treatment of mild respiratory failure with cycles of tripod position. Although more data are needed to draw definite deductions, this case suggest that in selected SARS-COV-2 patients, a better pulmonary blood flow distribution with improved V/Q matching can be obtained by shifting them in this position, thus avoiding the need for intubation and fasten recovery. We are aware that this represents a single success, thus providing only little evidence on safety and efficacy. But, compared to the risk related to intubation or prone position failure, in this single case, this position, associated to Helmet CPAP, has represented a valid alternative strategy.

## Data Availability

Data supporting our findings can be found at gilda.cinnella@unifg.it, the corresponding author.

## Abbreviations

COVID- 19: Coronavirus 2019
SARS-CoV-2: Severe Acute Respiratory Syndrome Coronavirus-2
CPAP: Continuous Positive Airways Pressure
PEEP: Positive End Expiratory Pressure
VE: Minute Ventilation
VT: Tidal Volume
RR: Respiratory Rate
V/Q: Ventilation to Perfusion Matching
mBP: Mean Blood pressure
HR: Heart Rate
FiO2: Fraction of Inspired Oxygen
SaO2: Oxygen Saturation
PaO2: Partial Pressure of Oxygen in Arterial Blood
PaCO2: Partial Pressure of Carbon Dioxide in Arterial Blood
PAO2: Partial Pressure of Oxygen in the Alveolar Gas
WBC: White Blood Count
CRP: C Reactive Protein
DIC: Disseminated Intravascular Cascade
IL-6: Interleukin-6
WOB: Work Of Breathing
FRC: Functional Residual Capacity
PP: Prone Positioning
ARDS: Acute Respiratory
ICU: Intensive Care Unit
